# Plasma modification in fruit juices: Changes in structure, colour, rheological parameters and sensory properties^☆^

**DOI:** 10.1016/j.fochx.2025.102445

**Published:** 2025-04-06

**Authors:** Anjaly Shanker Mundanat, Vipin Singh, Naveen Chandra Talniya, Sandeep Singh Rana

**Affiliations:** a Department of Bio Sciences, School of Bio Science and Technology (SBST), Vellore Institute of Technology, Vellore 632014, India; b Department of Catering and Hotel Management, School of Hotel and Tourism Management, Vellore Institute of Technology, Vellore 632014, India; c Department of Chemistry, Graphic Era Hill University, Dehradun 248002, India

**Keywords:** Colour, Plasma interactions, Rheological properties, Sensory scores

## Abstract

Cold plasma is an eco-friendly technology suitable for processing heat-sensitive food components. Compared to thermal treatments, the technique has the competence in delivering good quality, sensorial accepted, safe food products to the market. Consisting of a high-energy system of reactive species, plasma incurs interaction with the food components causing physical-chemical alterations, without employing any chemical agents to the system. Understanding system efficacy requires evaluating plasma effects on various food properties. The review focuses on the influence of plasma on the physical and sensory properties of fruit juices. Studies suggest the positive and negative inclinations in colour value of samples without deviating from acceptable range. Plasma has exhibited an ability to uphold the particle size distribution which in turn contributes towards the cloud stability, flow behaviour, and viscosity value. The taste, appearance, and mouthfeel of the plasma treated samples were superior than thermal treatments emphasising their value.

## Highlights

• Plasma treatment is advantageous over the traditional thermal treatment of fruit juices.
• Plasma efficiency is dependent on product characteristics and process parameters.
• Retention of colour and sensory properties of the samples has contributed towards the applicability.
• Particle size distribution, rheological parameters and stability of the juice were influenced by plasma application.

## 1 Introduction

Food dietary choices including fruit and fruit products was pursued for a long time owing to their nutritional and functional profile. Fruit juices are one of the popular segments accounting majorities of market share of fruit-based products. Worldwide clustered fruit juice segment has reported for a revenue gain of US$98,611 m in 2019 with an expected growth rate of 6.2 % from 2017 to 2025. The global market of fruit juices is expected to reach $90 billion by 2025 with a projected volume of 50.6 billion litres by 2024 (Agricultural and Processed Food Products Export Development Authority (APEDA), 2024). Fruit juices refer to non-fermented beverage fractions that are normally obtained by macerating or mechanically squeezing the fruits. The aforesaid demand indicates the need of suitable and efficient processing conditions that are involved in fruit juice preparation industrially. Thermal processing (TP) is the most common method in processing of fruit juices, which accounts for microbial stability to a greater extent. While the process accomplishes shelf-life stability of the product, it largely alters the nutritious, functional and sensorial profile (Ortega-Rivas & Salmerón-Ochoa, 2014). Higher temperatures are found to be negatively affecting the heat sensitive fractions like phenolic compounds, vitamins, odour- active components, pigments, and other bioactive. Alternates within the thermal processing segment like ultra-high temperature (UHT) and high-temperature short time (HTST) were found to be advantageous than TP, but the detrimental alternations were far away from the acceptable boundaries and good sensorial impression (Petruzzi et al., 2017). The search for alternatives to thermal treatment has paved the way for the upsurge of emerging non-thermal processing techniques. Deliverables as in product quality, safety and functional properties of food treated by these non-thermal techniques are significantly better than the thermally treated food products (Ortega-Rivas & Salmerón-Ochoa, 2014).

Non thermal processing of fruit juices with an aid of plasma technology has secured a wide relevance owing to its greenness, effectiveness, and lack of chemical residues. The principle of this technology involves the partial of total ionization of ions, reactive species, free radicals, charged particles in the form of electrons and photons, gas containing molecules, and quanta of ultraviolet and electromagnetic radiation (Surowsky et al., 2015). This gas ionization can be accomplished by the application of known energy source in the incidence of a neutral gas like air, nitrogen, oxygen, argon, helium etc. Rendered plasma system contains a plenty of charged particles (OH^−^, electrons, H_2_O^+^), reactive nitrogen and oxygen species (RNS & ROS including atomic oxygen & nitrogen, singlet oxygen, ozone, nitric oxide etc), excited molecules (excited N_2_ and O_2_), UV photons, free electrons and ions in total. This highly energized and interactive species of the plasma insinuates physical and chemical interactions in food components ensuring extended shelf life and safety. Proper understanding of these interactions plays a pivotal role in industrial application of plasma and this mechanism of action is highly dependent on the functional, biological or bioactive component present. Along with the product profile, these interactions are also dependent on several process factors like input power, gas constituents, gas type, gap between the discharge source and the sample, processing time, and the plasma source involved. A variety of plasma generating systems including dielectric discharge barrier discharge plasma (DBD), radiofrequency plasma, plasma jet (PJ), microwave (MW) plasma, glow discharge plasma etc. can be devised according to their intended final usage (Thirumdas et al., 2015). Among these, dielectric barrier plasma technique is the common plasma generating systems employed in food applications.

Suitability of plasma in controlling and managing the microbial populations in food is a subject that is widely studied and discussed (Shi et al., 2011; Wang et al., 2025; Xu et al., 2017). The mentioned array of reactive species including RNS, ROS, ions and other radicals have a contributory effect towards altering the microbial behaviour and response. This antimicrobial activity is attributed to the oxidative degradation of DNA proteins and cellular lipids weighing to intercellular damage of the system (Bourke et al., 2017). According to the reports, the technology is already successful in attaining efficient microbial reduction in cut fruits, whole fruits and even fruit juices/ pulp (Shi et al., 2011). The reports also state the possibility of interaction of these reactive species with the macronutrients and other bioactive components of food, leading to different chemical reactions viz. nitration, hydroxylation, oxidation, nitrosation, sulfoxidation, dehydrogenation or hydrogenation, deamidation, and dimerization. For instance, it is reported the presence of phenolic compounds in the fruit fractions get altered by the collective influence of plasma reactive species. Hydroxylation of benzene rings of phenolic acids is one of the widely accepted concepts, which leads to the formation of hydroxycyclo carbonyl radicals. Presence of these radicals initiates a cascade of reactions in the system leading to their effect in phenol profile. Another concept which is linked to the increase in phenolic content of CP treated food products is the catechin polymerization and increase in concentration of hydroxy cinnamic acids and chalones owing to these reactive species (Thirumdas et al., 2015). Suggestively, authors correlate the oxidation reaction and formation of reactive species as a byproduct of reactive interaction between the CP active species and different pigments (anthocyanins, carotenoids, betalains etc). Coming to the effect of plasma reactive species on macronutrients, reports include the effect of these interactive mechanism on ascorbic acid as well as lipid profile (Fernandes & Rodrigues, 2021). The highly reactive plasma species advocates the deprotonation of ascorbic acid leading to the formation of dehydroascorbate. Elevated ascorbic acid content is ascribed to cold plasma induced hydrogenation of dehydroascorbate. In case of lipids, active plasma species is considered responsible for the chain hydrolysis that exists in lipid-based compounds, leading to the reduction in saturated fatty acid content in avocado samples.

Feasibility of any thermal or non-thermal technique is closely related to the efficacy of the process considering its physical, chemical and biological attributes. CP treatment studies on fruit juices are mostly concentrating on the effect of the process variables on physicochemical parameters like pH, colour, total acidity, electric conductivity etc. and on chemical parameters like antioxidant activity, phenolic profile, ascorbic acid content etc. Along with these, researchers have also discussed about the feasibility of the technique in controlling the microbial populations in fruit juices. But the practical possibility of the technique is still ineffectual when we don't consider the physical properties like viscosity, rheological parameters, stability etc. and sensory properties into the picture. There are very rationed studies regarding the effect of plasma parameters on these variables and even the mechanism underlying the changes associated with these variables are practically unexplored. Understanding the feasibility of CP treatment requires evaluating its role in preserving juice fractions' physical characteristics, assessing the effects of plasma parameters on relevant variables, and identifying the limitations of plasma parameter ranges. Through this review, authors have made efforts to investigate and understand about the effect of plasma parameters on different physical and sensory properties of fruit juices in general.

## 2 CP treatment

**Fig. 1:**
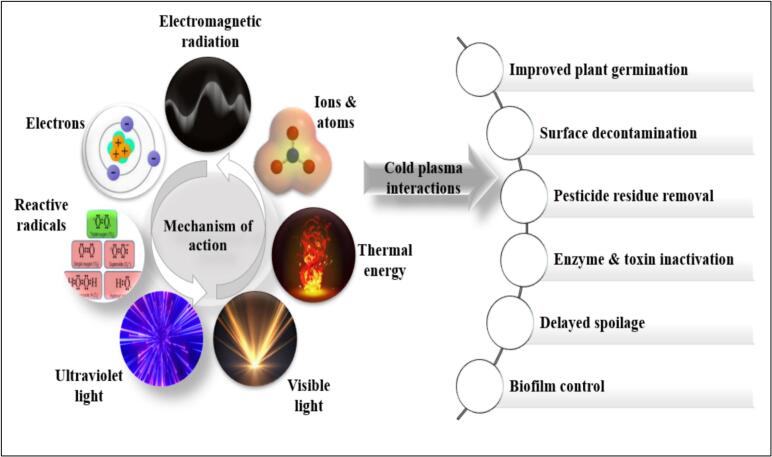
Summary on plasma constituents & applications of plasma interactions in food.

Plasma described as the fourth state of matter represents a high-energy system comprising charged particles, free radicals, unionized neutral molecules, ultraviolet radiation and a whole array of excited species (Fig. 1). Plasma generation basically gyrates around the ionization of gases which can be achieved by the application of different energy sources like electric field, thermal energy etc. right through a neutral gas (air, nitrogen, or oxygen etc.) resulting in dissociation and ionization of atoms (Thirumdas et al., 2015). Common gas system included in the process of generation are oxygen, nitrogen, and carbon dioxide. Besides the familiar sources, there is a discerned possibility of incorporation of noble gases like argon, helium or a suitable amalgamation of diverse gas sources (Coutinho et al., 2019). Selection of the operational gas is significant as it is essentially correlated with the voltage employed for the generation of plasma and also with the processing cost involved. Energization of this operational gas ensues the generation of reactive oxygen and nitrogen species, which in turn interacts with the food components and concludes with the obligatory alterations for safety and quality (Fig. 1).

Categorization of plasma systems established on temperature are thermal and non-thermal plasma. The former encompasses interactive species subsisting in thermal equilibrium and same temperature, while in the latter a non- equilibrium condition prevails. In non-thermal or cold plasma, the interactive species remains at an operated temperature range by controlling the energy reception and thereby promoting its wide range applicability in foods. Food processing and preservative applications involve different plasma generation technologies like dielectric barrier discharge (DBD), atmospheric plasma jet (APPJ), corona discharge (CD), glow discharge (GDP), microwave (MW), and resistive barrier discharge (RBD) plasma. Plasma generation technologies applied in to a system should be accomplished by keeping product characteristics in mind as it is one of the important indexes effecting the system efficacy (Fernandes & Rodrigues, 2021). DBD and APPJ are the most involved and reported plasma techniques in fruit juice treatments and are of utmost importance in the food and medical sector.

Dielectric barrier discharge plasma system is an economic feasible option where the plasma generation is executed by employing voltage between two metal electrodes. Positioned at a gap ranging between 0.1 mm to several centimetres, one or both the electrodes in the system is covered with dielectric material such as polymer, glass, quartz, or ceramic. Here, the presence of dielectric material aids in the generation of homogeneously dispersed plasma characterised by transient micro discharges. DBD plasma systems are designed and operated at an adapted range of voltages, frequencies, and gas pressures under varied gas sources (Fernandes & Rodrigues, 2021). This process is considered to be the most convenient form in food application due to its low cost, flexibility in electrode shape, dielectric material and configuration. In addition to the effectiveness of the system in fresh and packed produce, there are instances which show the efficacy of DBD plasma in in-package food treatment. Reported reduction in microbial counts by 2 log cycles were reflected in in-package decontamination of strawberry at a treatment period of 15 min (Rana et al., 2020). Plasma jet is an alternative production technology which involves several configurations in which plasma is shaped by another plasma source. The basic configuration of this setup includes a nozzle with two electrodes within which the carrier gases is passed by, and an application of high voltage electricity to this low-pressure gas instigates plasma generations. Oxygen, carbon dioxide, helium, or a mixture of ionizable gas fraction at higher frequencies and voltage can act as carrier gas in plasma jet system. This ionization and interactions will lead to the discharge of stable, uniform and homogeneous plasma which can be used in the treatment of food products (Thirumdas et al., 2015). Plasma jet treatment cycles were reported to be effective in 7.2 log reductions in microbial populations and displayed an improved quality profile than thermal treated juice fractions (Hou et al., 2019).

**Fig. 2:**
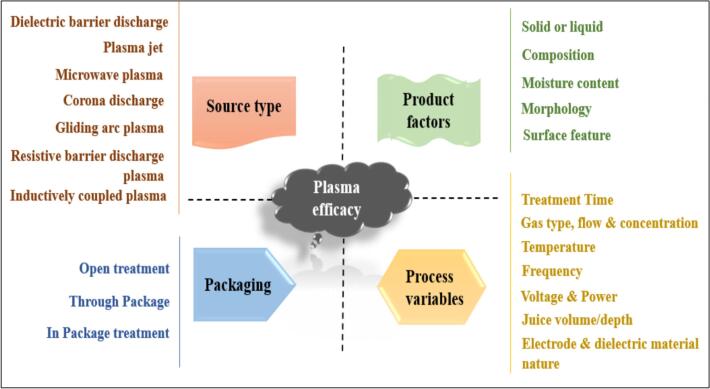
Brief representation of parameters influencing plasma efficacy.

As every other technique, the efficacy of the plasma application depends on different factors that are closely related to the system or the product that has been treated. A schematic representation of these parameters is given in Fig. 2. Each parameter considered has a major effect on the interactions that have been happening and thereby the usefulness of the treatment. For example, moderate treatment conditions were successful in maintaining the microbial loads and also in the retention of the functional profile of strawberry fruit. In-package atmospheric plasma treatment also ensured the storage stability of the produce for a period of 5 days (Rana et al., 2020). These interactions and usefulness vary from product to product, process to process, and also between the range of parameters. It is important to identify the type of plasma generation system, parameters involved and the applicable range of these parameters according to the product. Here, the process of modification in foods is basically centred on the interactions between the product and reactive species or components of generated plasma system.

## 3 Mechanism of action

### 3.1 Reactive species in cold plasma treatment

Plasma is often coined as ionized gas consisting reactive species in a metastable state and having a net zero electric charge (Thirumdas et al., 2015). The practicality of any plasma system is highly associated with the generated reactive species, their activity and further progressing reactions. The specificity of the reactive species in a plasma generator can be improved or altered with the application of different intermixtures of feed gases. Reactive oxygen (ROS) and nitrogen (RNS) are most prevalent and widely present reactive species in non-thermal plasma system. ROS is one of the dominating reactive species in all kind of plasma systems having extended lifetime. Attained from molecular oxygen, the set includes OH radicals, radical and non-radical oxygen, peroxyl radicals (ROO^−^), superoxide anion O_2_^−^ and non-radicals such as ozone, singlet oxygen, hydrogen peroxide etc. The reactivity, stability and penetration depth of each of these components varies and also is related to the product-plasma system involved. For example, ozone in known to exhibit highest oxidizing characteristics with highest reduction potential in liquid phase. Similarly, hydrogen peroxide is the significant reactive species formed during the plasma exposure in liquid systems and stability varies according to the pH variations. Coming to efficiency, the penetration depth and life time of hydrogen peroxide is more in comparison with other listed reactive fractions. Certain fractions like superoxide radicals etc. has a contributory effect towards furthering chemical reactions by acting as an intermediate. For the instance, superoxide take part in chemical reaction with nitric oxide forming peroxynitrite. This peroxynitrite is one of the species among RNS, which has an efficient diffusive and oxidizing nature. The others in the list of RNS are nitric oxide (NO), nitrogen dioxide, nitric and nitrous acid. Their formation and destruction are highly dependent on ROS as their origination involves the chemical interaction between excited nitrogen species like N and N_2_^−^ with the oxygen or other ROS like ozone, superoxide etc. Included in is nitric oxide, which is crucial intermittent accountable for the generation of other nitrogen reactive species. Nitrous and nitric acid of this group are long lived reactive species and has a causative effect towards the germicidal activity. Several experimental parameters correlated with the treatment like content of gas, properties of operational gas, type of plasma system involved, product characteristics and exposure time effects the interaction between reactive species and the food matrix. ROS and RNS fractions have an upper hand in microbial inactivation characteristics of plasma by damaging both cell membranes and intra cellular components and also reacting with macromolecules fractions in a microbial system. The penetration of cytoplasmic membrane of cells and their penetration is dominant feature that gives supremacy for ROS fractions in microbial inactivation.

### 3.2 Interactions and changes induced by the plasma reactive species

#### 3.2.1 Colour

**Fig. 3:**
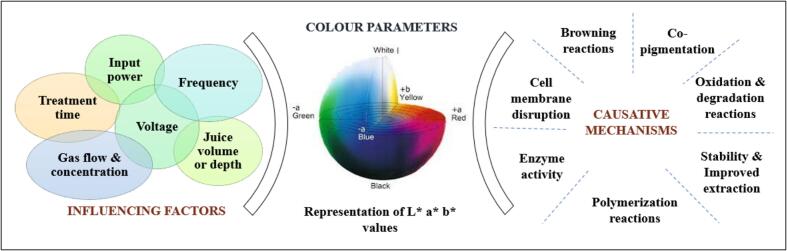
Pictorial representation of the mechanism involved, parameters evaluated and influencing factors in colour change.

Colour stability of processed foods goes hand in hand with the humane perception of good or high-quality food product. Variations in colour values of the different liquid formulations are entirely dependent on the plasma treatment method, properties of fruit and the process parameters. The positive and negative variations in sample colour can be associated with the structural, conformational or functional modification of different bio-actives or components of food product (Fig. 3). For example, in the case of pomegranate juice, the effect of atmospheric gas phase plasma is found to be very minimally or barely noticeable with a ∆E*^ab^ value ranging from 0.32 to 3.0 (Kovačević et al., 2016). The darkest colour difference or the darkest shade of the samples were observed in the case of control samples, which emphasize the contribution of plasma treatment towards the colour value of the juice sample. Here plasma reportedly aids in co-pigmentation of anthocyanins and other bio-actives like flavonoids, organic acids, alkaloids etc. contributing to the individual stability of these component fractions and thereby influencing the colour variations. Component co-pigmentation generates a typical conformational change in the chromophore group, increasing the absorptivity and an elevated shift of maximum visible wavelength ranges (Kovačević et al., 2016). These changes induce a darker reflection in anthocyanin colour formulating more intense colour for the sample. Comparable colour change results were reported in dielectric barrier discharge atmosphere cold plasma treated kiwifruit juice samples with the least colour variation reported at voltage of 20.4 kV, and time interval of 6.8 min (Kumar et al., 2023a).

Same parameter combinations of atmospheric plasma jet treatment showed varied results in orange and apple juices, which accentuate the implication of product component profile in the effectiveness of the treatment (Dasan & Boyaci, 2018). In the case of orange juice, the colour changes or ∆E value remained less than 3.0 throughout the treatment of 120 s, whereas that of apple juice was above 3.0 from the initial treatment periods. There was a possible decrease in the a* value or greenness of the orange sample after 30s of the treatment, while the b* value which represents the yellowness remained almost constant throughout. The variation was correlated with the isomerization of pigments and oxidation reactions. Possible explanations can be linked with the possibility of carotenoid pigment degradation by ozone and hydroxyl radicals of plasma species, leading to aromatic ring opening and advent of different aldehydes, ketones and organic acids. The reactive species of the plasma system can disrupt the cell membranes, releasing anthocyanin pigment to the extracellular space. This positively influences the intensity or measure content of the colour factor. Whereas, there is a degradation chance of anthocyanin fractions owing to prolonged treatment periods or high intensity plasma. Apple juices with noticeable colour change exhibited a visible darker colour values (L*) with progressing treatment. Redness value of the sample showed a slight difference with time with a greater difference in yellowness, mentioning the effect of browning reactions in the sample colour. The minimal colour difference in grape juice during high voltage atmospheric cold plasma technique was also networked with the degradation of ascorbic acid in the presence of reactive species and also to the progression of non-enzymatic browning in juice sample (Pankaj et al., 2017). The ascorbic acid degradation in presence of reactive species leads in formation of breakdown products like furfural, carbonyl compounds etc. Furthering chemical interactions of these component fractions changes or alters the colour of the food matrix. An analytical colour value related to the browning is a* and it is found that there is an association between treatment period and this reaction (Illera et al., 2019). Longer the treatment period, the lower the a* value, signifying a shift of sample colour towards greener lateral and reverses the browning effect. Degradation of anthocyanins and ascorbic acid was depicted to be the reason for the colour change in terms of variation in b* and a* values of plasma treated camu juice samples (de Castro et al., 2020). On a conflicting note, dielectric plasma system has reported inducing a higher ∆E values (more than 10) in apple juice for a treatment period of 0–40 s and power level ranging from 30 to 50 W (Liao et al., 2018). The treated sample exhibited a dark, greenish and yellowish tint with L*, a* and b* values decreasing with rise in parameter values under consideration. This noticeably colour change was elucidated to be in correlation with the polymerization of phenolic compounds that ensued with the incidence of plasma.

(L* = Lightness value, a* = Red-green value, b* = Yellow-blue value, ∆E = change in colour, kV = kilo volts, L/h = Litres per hour, ml/min = Millilitres per minute, dm^3^/min = Cubic decimetre per minute, Hz = Hertz, W = Watt)

Elucidation and summary on the effect of different process parameters on the efficiency of each plasma treatment method is included in Table 1. Each parameter displays a distinctive upshot in the final quality of the juice samples, and it is reliant on on the product and plasma process involved. There is a positive and quadratic effect of treatment time on the ∆E values depicting the colour change regarding the treatment process (Kumar et al., 2023a). Most of these colour changes associated with the treatment time are in a barely noticeable range, which exists without altering the overall acceptability of the final product. Parallel correlations were observed in the case of voltage implied in cold plasma treatment, where an increase in ∆E values was observed (Kumar et al., 2023a). Processing of sugarcane juice using the DBD plasma system also exhibits a similar dependency of colour over voltage, with the ∆E values not crossing a noticeable value range. Change in slightly noticed in case of L* value with voltage contributing to the variation in colour change (Manzoor et al., 2020). Final quality of the product during plasma treatment can also be correlated with the juice depth or to the total volume of the product (Kumar et al., 2023a). Lower depth of juice accentuates higher penetration capacity of plasma, inferring to low total volume and effecting the colour thereby.

**Table 1:** Effect of different process parameters in colour change and L*, a* & b* values of juice samples.

Sample	Process parameters	Results	Influencing mechanisms	Reference
Sugarcane juice	Dielectric barrier discharge plasma Voltage: 18–30 kV Time: 2 min	Slight decrease in L* value was noticed with an increase in voltage. ∆E values of the samples increased but remained within the noticeable range	Ascorbic acid degradation	Manzoor et al., 2020
Apple juice	Dielectric barrier atmospheric cold plasma Time: 0–40 s Input power: 30–50 W	Increase in a*& b* value and decrease in L* value with upsurge in treatment time. ∆E values increased consistently with increase in time and power	Polymerization of phenolic compounds and oxidation reaction of pigment components	Liao et al., 2018
Siriguela juice	Glow discharge plasma Time: 5–15 min Nitrogen gas flow rate: 10–30 mL/min	Elevated values of L* in untreated samples, with the highest value at 10 min of treatment. A reported rise in a* and b* values with slight dependency on time.	Pigment degradation by isomerization and oxidation reactions.	Paixão et al., 2019
Blended orange & carrot juice	Dielectric barrier discharge plasma Time: 5, 15, 30 s	Variation in L*, a* & b* value was noted with increase in treatment time. ∆E values increased above 3.0 value for longer treatment	Carotenoid pigment degradation in presence of ozone and hydroxyl radicals	Vukić et al., 2018
White grape juice	High voltage atmospheric cold plasma Time: 1, 2, 3, 4 min	Slight increase in a* value and b* value with increase in time	Degradation of ascorbic acid fractions leading to non-enzymatic browning	Pankaj et al., 2017
Orange juice	High voltage atmospheric cold plasma Time: 30, 60, 120 s	Increase in ∆E value with longer treatment periods but the highest ∆E values remained within the slightly noticeable range.	RNS and ROS initiated oxidation reactions	Xu et al., 2017
Blueberry juice	Cold plasma jet system- Oxygen & argon Gas content: 0, 0.5 %, 1 % of O_2_ Time: 2, 4, 6 min	Increase in L* value and a reduction in a* & b* value was noted with an increase in gas content and treatment time.	Oxidative degradation of anthocyanin owing to plasma generated species	Hou et al., 2019
Camu juice	Dielectric barrier discharge plasma Frequency: 200, 420, 583, 698, 960 Hz	Change in frequency doesn't affect L* value as such. Higher excitation energies (698 and 960 Hz) caused colour variations with change in a* and b* values, but not effecting the acceptability of the product.	Degradation of ascorbic acid and anthocyanin fractions	de Castro et al., 2020
Pomegranate juice	Atmospheric plasma jet system Time: 3, 5, 7 min Juice volume: 3, 4, 5 cm^3^ Gas flow: 0.75, 1, 1.25 dm^3^/min	Colour variation was significant in treated samples. No reported association with time and juice volume but showed a decline with increase in gas flow	Copigmentation of associated molecules	Kovačević et al., 2016
Apple juice	Atmospheric plasma jet system Gas flow: 3000 L/h Time: 30, 60, 90, 120 s	With an increase in treatment time, there is a reported decrease in L* and b* values, making the juice darker in colour	Ascorbic acid degradation	Dasan & Boyaci, 2018
Acerola juice	Nitrogen plasma treatment: Flow rate: 10–20 mL/min Time: 5–15 min	Flow rates and time caused increase in ∆E values up to 12 to 20 mL/min; 15 min treatment.	Polymerization of phenolic compounds and oxidation reactions	Fernandes et al., 2019
Orange juice	Atmospheric plasma jet system Gas flow: 3000 L/h Time: 30, 60, 90, 120 s	Slight decrease in b* values with a rise in time	Co pigmentation and oxidative degradation	Dasan & Boyaci., 2018

Hue (h*) and chroma (C*) are defined to be the qualitative and quantitative aspect of colour of food and food products. Chroma reflecting vividness or colour saturation is found to be on an increasing trend with the application of DBD plasma treatment of samples (Manzoor et al., 2020). This means that the instrumental colour of the sample becomes more vivid by the treatment of plasma in orange juice. Considering the hue of juice samples, there is a slight decreasing trend noted with the incidence of plasma. As the maximal decrease in the hue value is less than 2°, this deviation is on a minimal scale and doesn't affect the overall acceptability of the product in terms of colour values. Treatment times were reported to have a small difference in C* and h*, with higher treatment times showing a decrease in the values. Nitrogen glow plasma technique in acerola juice doesn't show much variations in chroma value regarding the time and flow rates, but displayed a difference in hue of up to 10 % increase with an intensification of time (Fernandes et al., 2019; Paixão et al., 2019). Parallel plot values as well as deviations from the above explained trends were seen in different sample products by employing a same plasma technique by under different dependent parameter values. There was a decrease in chroma value in apple juice, depicting incidence of greyer colour in the sample. Hue angle displayed an increasing or higher value, depicting the lesser intensity of red colour in apple juice. Decreasing trend is an indication of slight change in a* and b* values of the samples with plasma treatment. There is a major gap between the no of studies and different type of plasma system and parameter variations, which limits the possibility of any generalizations in the colour changes. There are variations in the mode and degree of reactivity of plasma species within the sample, as well among the different samples.

#### 3.2.2 Structure

Incidence of conformational or structural changes can modify the inclusive profile of a product, leading to functional and efficacy variation. The juice comprises two phases; one is the continuous phase and the other is the dispersed phase. Continuous or the serum phase consists of sugars, proteins, minerals etc. as intracellular components and the dispersed or the pulp phase consists of dispersed particulates, intact cells, tissues, and disruption residues from different employed processes. At what extent, the phase and how much change are induced in the system is dependent on the plasma method, parameter combinations and the product characteristics. Cold plasma is known and reported to have an effect on cell walls, causing the disruption and release of intra cellular components. The microstructure analysis of kiwifruit juice operated under DBD plasma system illustrates that cell integrity of plasma treatment was better than that of thermally treated samples depending on parameter combinations (Kumar et al., 2023b). Milder conditions of treatment were recorded to have a higher concentration of large particle sized components, substantiating the effect of plasma in particle size distribution. Intermediate treatment conditions upheld a comparable microstructure distribution with that of thermal treatments. Whereas the extreme treatment conditions were alleged to have more cell and tissue disruption and reveal the existence of agglomerates. Comparable results in the alteration of microstructure of liquid formulations were reported in the case of guava flavoured whey beverage (Silveria et al., 2019) and chocolate milk (Coutinho et al., 2019) emphasising the importance of parameter combinations in final quality. High-energy plasma system could have a more uniform, compact, non-aggregate and dispersion structure owing to the interactions of electrons, ions, and other plasma species hindering the formations of aggregates in the food system. Channelling high intensity plasma could result in surface damage owing to high electric current passed, but the particle size of the sample is reported to be small having irregular nature (Wu et al., 2018).

Secondary structural modifications were evaluated in plasma treatment of pineapple (Pipliya et al., 2024a) and kiwifruit juice (Kumar et al., 2024) illustrating the change in β- sheet, antiparallel β- sheets, β- turns, random coils and α-helix configurations. In comparison with the untreated control samples, there is an upsurge in β- sheet structure and diminution in the α-helix configuration owing to the plasma treatment. The change in the β- sheet configuration is in such a way that there is a decrease in β- turns and presence of random coils, while there is a reported increase in antiparallel β- sheets. Thermal treatment is expounded to have a reduction in β- sheet and α-helix configurations contained by the protein structure of juice samples. The changes attributed to plasma treated samples were linked to the presence of reactive species after the plasma treatment. The presence of reactive oxygen species directs to the oxidative degradation of proteins resulting in fragmentation, cross-link construction, unfolding and thereby contributing directly towards the conformational modification. The changes in structure are attributed to the covalent modification of protein by reactive oxygen species generated by cold plasma treatment and also by the interactions induced by the by products of oxidative stress. These conformational and unfolding inside the product matrix influences the functional profile of foods. Prevailing oxidative reactions stimulates the protein unfolding, resulting in increased surface hydrophobicity and compounded protein aggregation and polymerization. These structural modifications and their effect on functional properties are influenced by the range of parameters like treatment time and voltage (Eazhumalai et al., 2023). The unfolding of the structure is influenced by treatment time, when intermediate timings provide better degree of unfolding and thereby influencing the functional properties like foam stability and emulsifying properties (Segat et al., 2015). There is a helical content decrease observed with the increase in treatment voltage. The reason behind this change can be attributed to the formation of agglomerates at extreme treatment parameters, which in turn affects the flexibility and restricts the conformational rotation.

#### 3.2.3 Particle size distribution

**Fig. 4:**
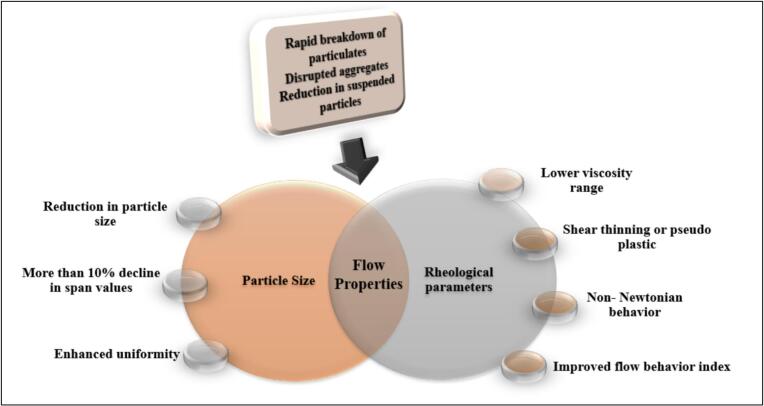
Summary on alterations happening in flow behaviour of juices regarding plasma treatment.

Particle size distribution of fluid mixtures is an important process control that affirms the claims related to the stability of the product. Information on the parameter also gives an idea about the flow behaviour of your homogenous liquid mixtures. The particle size distribution of fluid mixtures is found to be affected by the presence of plasma in the system and the extent of this effect can be correlated directly with the process parameters like time, voltage etc. Positive influence in particle size reduction of particulates was found in DBD plasma treatment with distribution range less than that of untreated sample (Kumar et al., 2024). The expanse and influence of the plasma system in the controlling the particle size of the liquid formulations needs to be investigated on a throughout scale (Fig. 4). This parameter variation can be correlated to the assumption regarding the oxidative characteristics of reactive species. Reactive fragments of generated plasma can oxidize organic compounds, potentially breaking larger compounds into smaller fractions. Illera et al. (2019) has documented the effectiveness in controlling the particle size of the samples owing to the plasma treatment. The author explains in detail about the time dependency of the parameter subjected to plasma system in cloudy apple juice. The particle size distribution of the samples showed an increased trend during the initial treatment period of one minute with an increase in D (Bourke et al., 2017; Coutinho et al., 2019) values followed by a decline in the particle size owing to 4- or 5 min treatment. Initial one minute of 10.5kv plasma treatment resulted in a change in d (0.9) value from 28.2 ± 1.2 to 45.5 ± 2. Variation in the particle size distribution values is narrow, concentrating differences in the bigger size ranges owing to the lower voltage treatments. The particle size variation can be ascribed to the coagulation of particles during the initial stages of treatment. The alteration in surface charge levels due to plasma treatment acts as a contributing factor to aggregate formation. Subsequently, the presence of reactive species induces oxidative and electroporating reactions, which ultimately result in particle size reduction. In addition, plasma treatment can denature the proteins leading to changes in their structure, size and thereby influencing the overall particle size distribution (Eazhumalai et al., 2023). Increasing the plasma treatment time resulted in a decrease in particle size where the values of d (0.9) came down to 7.8 ± 0.1 compared to the untreated sample values ranging at 28.2 ± 1.2. Span values representing the degree of consistency and distribution width also reported a similar trend of increasing value followed by decline in value with the longer treatments. After 5 min treatment, there was decline in span value from 106 ± 7 in the untreated sample to 28 ± 2 in the treated one, indicating better homogenised samples at the end of the treatment. Lesser distribution width or band value always corresponds to the homogenization of the liquid samples, contributing to the stability. Positive inclination towards the stability was noted in the case of pineapple juice treated with non-thermal plasma system (Pipliya et al., 2024b). Apparent reduction in sauter mean diameter from 1617 to 894 nm and volume mean diameter from1688 to 917 nm ensured the stability of the juice fraction post treatment. Substantial reduction in the suspended particles and higher uniformity in the lower span values by plasma treatment contributed to the stability factor. Span value of the optimized plasma treated samples were observed to be better and lower than thermal treatment, mentioning the stability factor (Kumar et al., 2023a). This uniformity is in turn expedient in making the liquid formulations more appealing to consumers by increased mouthfeel and sensory attributes. Analogous reports about voltage dependency in particle distribution were reported in wheat sprout juice owing to DBD plasma treatment (Manzoor et al., 2023). Plasma interactions caused a reduction in particle size at higher voltage treatments and augmentation in volume of particulate matter at lower voltage.

Considering cold plasma as an alternative to thermal pasteurization emphasize the need for understanding how these techniques affect the particle size and distribution. Thermally pasteurized sugarcane juice samples were found to be having higher no of large particles compared to DBD plasma treated samples at 30 ± 2 °C and 30, 35, 40, and 45 V for 2 min (Manzoor et al., 2020). The author has reported a significant decrease in D (Bourke et al., 2017; Coutinho et al., 2019), D (Agricultural and Processed Food Products Export Development Authority (APEDA), 2024; Bourke et al., 2017), d (0.1), d (0.5), d (0.9) values in sugarcane juice samples owing to DBD treatment whereas all the parameters have higher values for thermally processed juice samples. The influence of voltage values in the distribution is reported in the study, with lower voltages promoting higher particle sizes and a decrease in the particle size distribution with increase in voltage value. Analogues to the former study, the span values are also in correlation with the recorded parameter values. Distribution width of thermally pasteurized sugarcane juice samples ranged at 23.51 ± 0.35 while that of plasma treated samples was at 4.03 ± 0.04. Better homogenization achieved through cold plasma treatment of sugarcane juice samples contributes to the better stability and shelf-life retention of samples. DBD plasma treated sugar cane juices are suggested to follow a monomodal distribution, which affirms the stability parameter of the sample. Particle distribution width defined as span value appears to follow a similar trend in comparison with thermally treated pineapple juice samples, contributing to delivering a better and homogenised end product (Pipliya et al., 2024b). With the limited studies on the variations in particle size distributions of fluid samples, optimization and generalizations on treatment parameters remains as an ardours task and needs more investigation in detail.

#### 3.2.4 Cloud change & stability

A poor cloud stability implies progress of sedimentation in liquid formulations, leading to negative influence on freshness and consumer experience. There are many factors that affect the cloud stability of juice formulations including size/distribution of particle size, activity of enzyme fractions like pectin methylesterase and even the presence of pectin. While correlating the parameter with plasma, an anticipated positive trajectory can be flaunted with the known effect plasma has on particle size distribution. The span value of the sample indicating the distribution width was comparatively lower in the case of optimized plasma treatment, which emphasizes the even distribution of particles (Kumar et al., 2024). This homogeneity factor within the juice samples is contributory towards the cloud stability. Cloud value of the samples was sustained post plasma treatment in cloudy apple juice and showed a decreasing trend with respect to storage time (Illera et al., 2019). Here the author correlates cloud value variation to the electrostatic repulsive force caused by the demethylated protein within the samples which hinders the particle coagulation. The decline in this cloud value with storage was sooner in samples with the shortest treatment times, and an optimum value of 0.18 was obtained for 5 min treatment after 28 days of storage. A parallel course of change was observed in cloud stability, which showed a decrease in value with storage period. Along with the particle size distribution, different interactions between components like proteins, pectin, polyphenols etc. during and after the treatments normalizes sample's course of path pertaining to cloud stability. Vukić et al. (2018) correlates the change in cloud value of orange and carrot juice blend to the possible inactivation of pectin methylesterase enzyme. Shorter treatments showed a positive influence on turbidity value and thereby contributing towards cloudiness of the samples. In addition, the polymerization and oxidative reactions of phenolic fractions, pectin, and organic compounds might be a causative agent in retention of parameter value. Prolongation of treatment strategically decreased the value, with final treatment combinations exhibiting a turbidity value comparable with untreated sample. The parameter needs to widely investigated in terms of plasma system and treatment combinations to get a better idea on how and why the variation is happening.

#### 3.2.5 Rheological properties

The notion outlining the phenomenon of deformation and characteristic flow of fluid owing to external forces compasses the study of rheological behaviour of food materials. The parameter gives an idea about the flow behaviour or pattern of your food material and in the case of fluid foods, it is measured by evaluating the shear stress vs shear rate. Plasma treatment of fruit juices involves an advancement in non-Newtonian pseudo plastic behaviour with a variation in consistency index (*K*) of the samples with the treatment (Manzoor et al., 2020; Pipliya et al., 2024a). Pseudo plastic behaviour of the juice samples decreased with CP treatment in comparison with the thermal treated samples, showing their inclination towards the idea flow characteristics (Kumar et al., 2024). There is a noted decrease in apparent viscosity with an increase in shear stress applied to the samples. This decrease in viscosity can be attributed to the rapid breakdown of the particulates and suspended particles owing to the initial shearing. Increase in shear rates to a particular extent corresponds to structural conversions like disrupted aggregates in the samples steering to smaller particulate conformity. Diminished particle size contributes to lesser consistency, lower apparent viscosity and enhanced flow properties (Fig. 4). Additionally, the role of protein and pectin modifications and the breakdown of polysaccharides by reactive species like ozone or hydroxyl radical could act as key component. The reactive fractions ensure the decrease in complexity of these components and this reduction will reduce the viscosity values, directly impacting the flow properties.

A similar inclination in shear rate and apparent viscosity variation was noted in case of wheat sprout juice (Manzoor et al., 2023), chocolate milk (Coutinho et al., 2019) and guava flavoured whey beverage (Silveira et al., 2019) with reduced apparent viscosity and consistency index than thermal treated or untreated juice samples. The reduction of apparent viscosity was also observed in the case of guava flavoured (16 % guava pulp) whey beverage, with an increase in the shear stress and lower consistency index. Here, the author mentions a linear relationship between the cold plasma processing conditions and the changes in viscosity of the whey beverages (Silveira et al., 2019). Corresponding impact of decrease in stress, consistency, and flow behaviour index was reported in guava flavoured whey beverage with an attributed change in treatment time and gas flow rate. Milder conditions (low treatment time and gas flow) constituted higher values of variables, and an increase in parameter values caused a reduction in enlisted variables under consideration. Severe processing conditions tend to increase the viscosity of the whey beverages, while lower and mild processing conditions account for lower viscosities of the sample. Viscosity followed an increasing trend with an increase in voltage, treatment timings, and with an increase in flow rate. There is an initial upsurge in shear stress value, followed by a diminution and a final surge in value regarding increasing voltage. Consistency index also shows a parallel variation in value with an increasing voltage, while apparent viscosity of the samples follows an unvarying declining trend (Manzoor et al., 2020). Similar inconsistency was noted in the case of wheat sprout juice owing to voltage variation in DBD plasma systems (Manzoor et al., 2023). At the same time, there is a decline in shear stress and consistency index of pineapple juice pertaining to increase in voltage in DBD plasma treatment system. This inconsistent difference can be attributed to the characteristic profile of the sample and also to the interaction happening within the product, plasma system and parameter combinations. Synchronization of these substantiations can be done in terms of plasma systems, similarity of product and its characteristics and also according to the process parameters.

Parallel to these findings, there was an increase in the viscosity of mango pulp treated by dielectric plasma system during the initial period of the treatment. The viscosity values of the sample displayed an initial upsurge up to a treatment period of 6 min, trailed by a decrease in the value with the rise in time. Treatments at 8 min and above has reported to be following a former trend of viscosity decrease (Abedelmaksoud et al., 2022). The initial rise in the parameter value was ascribed to the inactivation of pectic enzymes present in the sample due to the plasma activity. Storage stability of the juices in terms of viscosity has increased with the application of plasma. There is a positive inclination which shows reduction in viscosity rise of the sample and is correlative to the inactivation of pectin methylesterase enzyme by the application of plasma (Nasri et al., 2023). In addition to this change in viscosities of the samples by plasma treatment is also linked to the potential reductions in molecular weight and altered hydration properties (Eazhumalai et al., 2023). There is a material variation that is largely contributing towards the change in the parameters under investigation, and the comprehension of the influencing factor for different types of food product needs quite an amount of effort and time.

Frequency sweep approach measurements made over a range of parameters exhibited an inclination of storage modulus (G') and loss modulus (G") values regarding the plasma treatment. Although there is only rationed information on how the range of different parameters affect the elastic and viscous behaviour of the product, the normal tendency of some liquid formulations is discussed. Over different frequency ranges, there is an upward increasing trend in storage G' and loss G" modulus that is followed in cold plasma treated and thermal treated samples of pineapple juice. The most noticeable G' and G" values were reported in thermally treated samples followed plasma treated samples and untreated pineapple juice. Variation in elastic behaviour of the juice is attributed to the plasma effect on the particle morphology, aggregation, and flexibility related to a varied particle distribution. The breakdown of proteins, polysaccharides, and pectin molecules in the presence of reactive species may play a role in modifying the viscoelastic properties of the juice, thereby contributing to changes in the storage modulus (G') and loss modulus (G"). In some cases, plasma treatment can induce cross-linking of certain molecules, which might increase G' and G", depending on the juice composition and treatment parameters. Frequency increase has resulted in the increase in both modulus values, with thermally treated samples showing higher variation between storage and loss modulus. Plasma treated samples maintain minimal difference between both modulus values exhibiting elastic and viscous nature of the samples. Plasma treated kiwifruit juice samples follows a comparable course with higher deviation between the modulus values were obtained in the case of untreated followed by thermally treated ones. Analogous modulus values were obtained between plasma extreme and optimized treatments, and this change corresponds to the explained flow pattern of the sample. Parallel results in the influence of frequency are reported in the case of ultrasound treatment of kiwifruit juice, and the results elucidate a higher storage modulus value in comparison with G" at the frequency range of 0.1-10 Hz (Wang et al., 2020). Temperature, pressure, composition, and time were also described as influential parameters in ultrasound and high-pressure homogenization of different fruit juice samples (Huang et al., 2018; Wellala et al., 2020).

#### 3.2.6 Sensory parameters

**Fig. 5:**
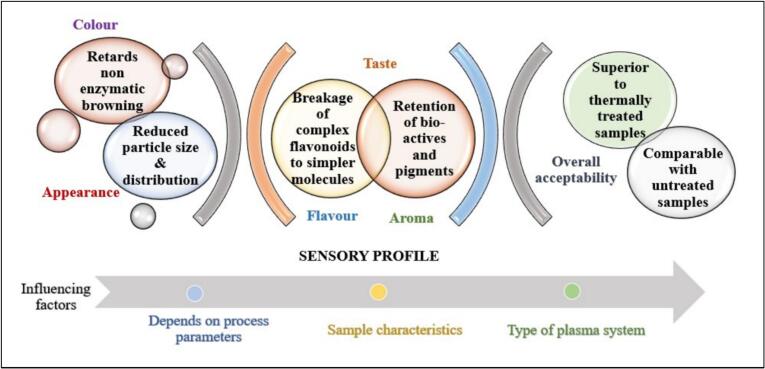
Sensory parameters of fruit juices: Factors & parameters under consideration.

The major organoleptic and sensory parameters of fruit juices under customer consideration are colour, flavour, aroma, and taste. The influence of processing method on any of these parameters directly stimulates the product's sensorial data, which in turn affects the acceptability of the product (Fig. 5). While colour and appearance display a primordial effect on the perceptive quality, flavour and taste have the major command on acceptability and consumption of any fruit or related products. Fuzzy logic studies on sensorial data of plasma treatment reveals the comparable nature of process treated samples with the untreated ones. The taste and mouth feel of plasma treated samples were appreciated, emphasising the retention of bioactive and nutritional fractions in the kiwifruit juice sample. Particle size reduction owing to the CP treatment contributes towards the overall acceptability of product with similar standard fuzzy scale values of treated and untreated product fractions (Kumar et al., 2024). Thermal treatment data showed an analogous effect of inferior data set values, owing to the non-enzymatic browning and pigment loss at higher temperatures. Sensorial parameters of cold plasma treated samples surpassed the thermally treated fractions, with parameter scale values under acceptable thresholds and sensorial stability over prolonged storage period (Pipliya et al., 2024a). Retentive and superior acceptability scores of cold plasmas over thermal treatments was ascribed to the conversion of complex flavonoids fractions into smaller and simpler compounds owing to plasma and thereby leading to flavour enhancement. Retention of orange-like aldehydic descriptors in glow discharge plasma treated juice ensured more developed aroma, further maintaining the citrus descriptors as primary odour (Rodrigues & Fernandes, 2022). Plasma treatment was also helpful in mitigating the off-flavour development caused by the α- terpineol and thereby improving the flavour of the product. Flavour enhancement is attributed mainly to the interaction of reactive oxygen species leading to the oxidation or modification of volatile compounds. Another causative factor could be the influence of plasma in sugar and organic acid levels of juice fractions. These interactions can alter the structure or presence of these compounds, and thus affect the sweetness or tartness of the product. Polymerization and oxidative modification of phenolic compounds can also affect the level of astringency or bitterness in juice, which in turn contributes towards the overall flavour and acceptability of the product.

Like all other discussed parameters, the sensorial behaviour of the juice samples was also affected by the parameters involved. Extreme plasma techniques negatively affected the sensorial scores of the juice samples and exhibited a value comparable with thermal treatments. Longer duration and juice depth treated at lowest depth has less acceptance and sensory scores compared to the optimum treated kiwifruit juice samples which exhibited a sensory score matching very good or excellent criteria (Kumar et al., 2024; Pipliya et al., 2024a). Application of plasma has contributed towards the enhancement of aroma intensity of samples safeguarding the aroma profile (Maia et al., 2023). There was a reported 28 % increase in aroma intensity of the samples with notable changes in the fatty acids and fatty acid ester concentration. Plasma reactive species could be responsible for the internal segmentation of explained fractions, leading to the development of more acceptable pleasant and fruity aroma. On top, formation of alcohols due to hydrogenation of aldehydes in the system also contributed towards an improved aroma profile. The treatment times were found to be beneficial in ensuring the aroma profile development as the plasma effect for an extended time likely enhanced the concentration of volatile like aldehydes, short chain esters etc. Moderate frequency levels have a contributory effect on the fruity aroma and also the concentration responsible volatile components, while higher frequency levels intensify the effect of sulfurous descriptors (Porto et al., 2023).

## 4 Challenges, limitations, and safety aspects

Cold plasma technique is such a unique, sustainable and potential option which can be focused on food safety along with the quality enhancement (Table 2). But there is a certain ambiguity that prevails in different aspects. Cold plasma applications need a throughout investigation on the nature and types of reactive species generated and about their interactions. It is not that there are no studies associated regarding the subject, but it's important to understand whether there is any difference in reactive species nature or intensity with change in plasma generating system, configurations and according to the carrier gas. If there is a difference, we need to characterize it as it directly impact the efficiency of the system. Reactive species nature is communicated to be responsible for shaping the food interactions, decontamination efficacy and other functional changes. Along with the study on interactive species, there is a need to understand the extent of changes that this process induces on the product and how the change in reactive species causes changes in the physicochemical and safety profile of food. From a regulatory perspective, the consent for a new plasma process needs a substantial volume of data collection, analysis, and time. The assessed values of these investigations give a roadmap of approval pathway of plasma process and thereby aids in gaining consumer acceptance. Largely, consumer acceptance towards new and innovative technologies is a bit unclear as they perceive them as unknown and riskier than traditional practices. The ambiguity of the process among the consumer population can be eliminated by increasing the public awareness and generating public opinion data. Educating consumers about the economic, environmental and benign effects of cold plasma on food should help in this process.

**Table 2:** Summary table on the influence of plasma on different characteristics of fruit juices.

Sample	Plasma system	Process parameters	Results	Reference
Sugarcane juice	DBD	Voltage: 18–30 kV Time: 2 min	• Decrease in L* value and increase in ∆E values • Reduction in particle size • Non-Newtonian pseudo plastic behaviour • Apparent viscosity of samples decreased with rise in shear rate	Manzoor et al., 2020
Blended orange & carrot juice	DBD	Power: 24 W Time: 5, 15, 30 s	• Lighter in colour • Variation in L* value and a decrease in a* & b* value • Slight increase in juice turbidity	Vukić et al., 2018
Siriguela juice	GDP	Gas flow rate: 10–30 mL/min Time: 5–15 min	• Change in a* and b* values • Hue & chroma values exhibited a small change	Paixão et al., 2019
Kiwi fruit juice	DBD	Voltage: 30 kV Time: 6–10 min	• Lower consistency index & apparent viscosity • Pseudo plastic behaviour • Colour retention of the sample with ∆E values within the acceptable range • Sensory attributes were better for lower time	Kumar et al., 2024
Pineapple juice	DBD	Voltage: 20 kV Frequency: 50–1000 Hz Time: 10–20 min	• Enhancement of fresh and fruity descriptors of aroma profile • Treatment at 50 Hz reduces undesired aroma, promoting the sensory profile • Contributory effect on the fruity aroma and also the concentration responsible volatile components	Porto et al., 2023
Apple juice	GDP	Voltage: 20 kV Frequency: 20–65 kHz Time: 1–5 min	• Best treatment: 10 kV for 5 min • L* values of 5 min treatment was better than one min; all within acceptable range. • Colour stability was maintained during storage; better stability for 5 min treatment • Increase in treatment time decreased the particle size • Quality of juice was maintained during the shelf-life period of 21 days	Illera et al., 2019
Pineapple juice	DBD	Voltage: 38–45 kV Time: 631–900 s	• Marked reduction in particle size and consistency index • Reduction in sauter and volume mean diameter signifying stability of treated ones • Indication of pseudo plastic behaviour • Comparable sensory values with untreated samples	Pipliya et al., 2024a
Guava flavoured beverage	DBD	Gas flow rate: 10–30 mL/min Time: 5–15 min	• Exhibited lower viscosity and low consistency index • Flow behaviour index similar to Newtonian fluids; pseudo plastic behaviour • Extreme conditions: high no of larger particles and less smaller particles	Silveria et al., 2018
Orange juice	GDP	Gas flow rates: 10 to 30 mL/min Time: 10 to 30 min	• Retention of orange-like aldehydic descriptors • Ensured more developed aroma & contributing towards sensory profile • Reduced off flavours by 61 %	Rodrigues & Fernandes, 2022
Sugarcane juice	DBD	Voltage: 30–45 V Frequency: 10 kHz Time: 2 min	• Reduction in particle size • Reducing trend in particle size distribution with increasing voltage; exhibits monomodal distribution • Non- Newtonian pseudo plastic behaviour • Reduction in apparent viscosity values and consistency index	Manzoor et al., 2020

Another important obstacle in the route of practical applications of plasma is the development of user-friendly, low maintenance plasma equipment tailored for different types of fruit juices. While there is a segment of consumers willing to pay a premium for high-value products, the broader population continues to seek cost-effective and healthy alternatives. This trend exerts pressure on the economic feasibility of balancing affordability with quality. Developing plasma systems that are cost-effective, efficient, suitable for large scale applications with product generality and cost-effective stills remains as a challenge. Focussing through research, following by prototype development, pilot scale testing and commercial application with open innovation through academia-industry-government collaboration can help in the growth and increased application of cold plasma in food systems. When proposing for large-scale applications, the health and safety of the process and operating system needs to be studied along with the engineering design. Within health and safety of the process, a no of factors comes to play. A notable one among them is the equipment and personnel safety. Higher concentration of reactive species and high voltage is two major factors that is correlated with the equipment and worker safety concerns. Exposure to such a hazardous combination of reactive species is harmful to the working personnel and needs ample procedures for chemical safety installed in place. Carbon filters, leakage sensing devices and good ventilation standards ensure and act as a proactive measure. Another one concern related to the safety perspective is the presence of chemical residues or secondary compounds. The plasma process generates reactive species that can interact with food components to form potentially harmful secondary compounds. The extent of this phenomenon is largely influenced by the specific plasma process, parameter settings, and product characteristics. While the possibility of such risks cannot be avoided, these issues can be effectively managed through the careful selection of processes and parameters tailored to specific food product groups. This highlights the importance of ongoing research into plasma processes to enhance understanding and ultimately eliminate potential risks

## 5 Conclusion & Future prospects

Cold plasma technique is an efficient, innovative and promising technology in prolonging the shelf life and upholding the quality of fruit juices. The recent years of the study around plasma was concentrating mainly on its microbial efficacy, degradation potential of pesticides, toxins etc. and also in maintaining the nutritional and chemical potential of the treated samples. Plasma treatment, as a non-thermal processing technique, stands out for its versatility, rapidity, efficiency, adaptability, and eco-friendliness. This low-temperature method is particularly suited for sensitive food components across various phases and surfaces, including solids, liquids, and irregular structures. In comparison, technologies like ultrasound and pulsed electric fields exhibit limitations in their scope, particularly when it comes to the diversity of surfaces and phases they can effectively treat. Moreover, plasma treatment showcases superior microbial efficacy, demonstrating higher efficiency and a broader applicability across various microbial species, giving it a significant advantage over these alternative methods. This highlights the importance of further development and research into the applicability of this technique. However, the effect of plasma treatment on the physical properties of fruit juices is a minimally investigated and needs more attention. Aligned with other parameters, the influence of reactive species in the physical properties of fruit juices is significant. The possible mechanism behind the variations encircles co-pigmentation, oxidative degradation and polymerization reactions happening under the influence of these reactive species. The degree of this influence is related to the process as well as product characteristics. Those studies that involve the possibilities of different range of parameters and different product specificity are still in nascent stage. In the coming years, extensive research opportunities should emerge around process-product interactions, cultivating more profound understanding and thereby enhancing the applicability of plasma technology. Future possibilities should also explore the potential of combination of plasma technology with other food processing technologies like ultrasound, pulsed electric field etc. For instance, the combination of reactive plasma species with the cavitation effect of ultrasound aids in eliminating pathogens from the system. Similarly, the complementing antimicrobial properties of techniques like plasma and high-pressure processing would ensure high safety standards. These combinations still being explored have a potential in reforming food processing techniques, addressing the increasing consumer demand for high-quality and safe products.

## CRediT authorship contribution statement

**Anjaly Shanker Mundanat:** Writing – review & editing, Writing – original draft, Visualization, Supervision, Resources, Methodology, Formal analysis, Conceptualization. **Vipin Singh:** Writing – review & editing, Methodology. **Naveen Chandra Talniya:** Visualization, Resources, Methodology. **Sandeep Singh Rana:** Writing – review & editing, Supervision, Formal analysis, Conceptualization.

## Declaration of competing interest

The authors declare that they have no known competing financial interests or personal relationships that could have appeared to influence the work reported in this paper.

## Data Availability

No data was used for the research described in the article.
